# Beneficial effects of Heqi san on rat model of polycystic ovary syndrome through the PI3K/AKT pathway

**DOI:** 10.1186/s40199-017-0188-7

**Published:** 2017-10-11

**Authors:** Hengxia Zhao, Daocheng Zhou, Ye Chen, Deliang Liu, Shufang Chu, Shimao Zhang

**Affiliations:** 1 Department of Endocrine, Traditional Chinese Medicine Hospital of Shenzhen, Shenzhen, Guangdong 518033 China; 2 Longhua Central Hospital of Shenzhen, Shenzhen, Guangdong 518000 China

**Keywords:** Polycystic ovary syndrome, Heqi san, PI3K/AKT pathway, miRNA PTEN

## Abstract

**Background:**

Heqi San, a traditional Chinese medicine (TCM) has been reported to regulate hormone levels in patients with metabolic disease, suggesting a potential clinical application. In the current study, we aimed to elucidate the effect of Heqi San on rat model of polycystic ovary syndrome (PCOS).

**Method:**

PCOS model was established in female SD rats. Rats were randomly divided into four groups: the control, untreated PCOS model, Heqi San treated PCOS model (8.1 g/kg) and metformin (MET) treated PCOS model (135 mg/kg) groups. All animals were subcutaneously injected with 6 mg/100 g dehydroepiandrosterone (DHEA) in the neck once a day for 20 consecutive days. The serum hormone levels were measured by ELISA. The ovarian tissues were stained with hematoxylin and eosin (HE) to undergo pathological examination. The expression levels of GLTU4 and PTEN mRNA were examined by real time PCR. The crucial proteins in the PI3K/APT pathway were analyzed by western blotting. Then, the functions of the target genes were analyzed using bioinformatics approaches.

**Results:**

We found that Heqi San was able to recover the serum hormone levels and improve insulin resistance in PCOS rat model. A morphological lesion of the ovary was also restored with the Heqi San treatment. More importantly, we discovered a correlation between the PI3K/AKT signaling pathway and the beneficial effects of Heqi San, demonstrating that its application could alter the expression levels of p-ERK, p-AKT, p-GSK3β, IRS-1, PTEN and GLTU4, all key factors in the PI3K/APT pathway. Through a bioinformatical analysis, we predicted the related gene function and pathway of the pathological mechanism of PCOS and found miRNAs that are likely to be critical in PCOS occurrence, including rno-miR-144-3p, rno-miR-30c-2-3p, rno-miR-486, rno-miR-3586-3p and rno-miR-146b-5p.

**Conclusion:**

The beneficial effects of Heqi on PCOS, including alter serum hormone levels, recover ovary morphological lesions and improve insulin resistance, which is mediated through the PI3K/AKT pathway.

**Graphical abstract:**

The potential role of miRNA-144-3p in PCOS pathogenesis.
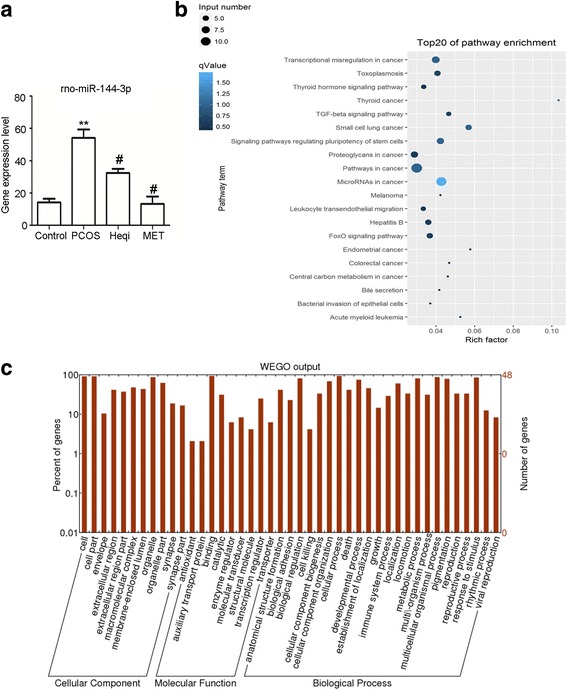

## Background

Polycystic ovary syndrome (PCOS) is a common and multifactorial disease associated with both endocrine and metabolic disorder. It affects approximately 4%–18% of all reproductive-aged women in the world [1, 2]. PCOS is characterized by hyperandrogenism and ovarian abnormalities, resulting from a disruption in the hypothalamic–pituitary–ovarian axis [3, 4]. Clinically, the main cause for reproductive and metabolic abnormalities in women with PCOS are hyperandrogenism and insulin resistance [5]. The etiology of PCOS is still unknown, although environmental, genetic, and hormonal factors are all thought to be important in its development [6].

Since PCOS has clinically heterogeneous characteristics, its treatment is complex and elicits variable responses among PCOS patients [7]. One of the most widely used medicines for PCOS treatment is metformin, an insulin-sensitizing drug [8], which can increase the insulin sensitivity of ovaries to enhance glucose uptake [9]. Additionally, other types of medicine have also been applied to PCOS. For example, 3-iodothyronamine was found to reprogram lipid metabolic pathways [10], and soy isoflavones had beneficial effects through the inhibition of aromatase activity [11]. Furthermore, physical therapy, such as acupuncture, has been shown to be effective in improving the insulin resistance of PCOS patients [12].

In China today, traditional Chinese medicine (TCM) is often administered as a complement to western medicine. For example, a common preparation used to improve sexual performance in men with sexual and erectile dysfunction is *Curculigo orchioides Gaertn.* [13]. TCM has recently been reported to exert clinical effects in PCOS treatment. *Origanum majorana* tea may regulate hormone levels in women with PCOS [14]. *Aloe* has been shown to restore lipid profiles in a PCOS rat model [15]. The biochemical and clinical parameters of a PCOS rat model were improved with chamomile extract treatment [16]. Furthermore, *Labisia pumila* (Myrsinaceae), known as “Kacip Fatimah”, was demonstrated to have beneficial metabolic effects in a PCOS rat model [17]. Taking the limited side effects of traditional herbal medicine into consideration, all of the above data demonstrate the potential role of TCM in the management of PCOS. Some of the physiological mechanisms behind the efficacy of most TCMs are still unknown for PCOS, partially due to the fact that most TCMs contain multiple components, although some have been proposed. For example, *Salvia miltiorrhiza Bunge* has been shown to significantly improve glucose tolerance in a prenatally androgenized rat model of PCOS [18]. *Rhizoma coptidis* and *Ocimum basilicum* induced ovulation through estrogenic effects when used to treat PCOS [19].

*Curculigo orchioides Gaertn.*, *Cuscuta chinensis Lam.*, and *Poncirus trifoliata (L.) Raf.* have commonly been used to treat impotence [20], *Cynanchum otophyllum C. K. Schneid.* has been proven in Chinese literature to replenish the blood [21]. Traditional Chinese physicians believed that the basic pathology of PCOS is kidney deficiency and blood stasis. Thus, the TCM treatment for PCOS mainly focuses on how to remove blood stasis [22]. The basic philosophy of Heqi San for PCOS treatment lies in nourishing the liver, kidney, spleen, and blood, and in promoting blood circulation to overcome stasis, supplement “qi”, and nourish “yin”. In addition, the flavor theory of this TCM is “Gan, Wen, and Ziyin”, which has been shown to be safe in PCOS therapy [23].

In this study, we demonstrated the application of Heqi San, a traditional Chinese medicine (TCM) in a PCOS rat model. Heqi San has been reported to have beneficial effects on the treatment of diabetes and other metabolic diseases, making it likely to function as a candidate for the treatment of metabolic disease in PCOS.

## Methods

### PCOS model

Forty female Sprague–Dawley (SD) rats (aged 3 months, weighting 300 ± 20 g) were obtained from the Laboratory Animal Centre of Guangzhou University of Chinese Medicine (Guangzhou, China). Animals were kept in groups with free access to food and water and a controlled temperature of 22 ± 2 °C with a 12 h light/12 h dark cycle. All rats used in this study had normal estrous cycles prior to treatment, as confirmed by examination of vaginal smears under a light microscope for two sequential cycles (about 8–10 days). The PCOS rat model was established according to a previous study [24]. Briefly, the animals were subcutaneously injected with 6 mg/100 g dehydroepiandrosterone (DHEA) in the neck once a day for 20 consecutive days. Rats were randomly divided into four groups (*n* = 10 per group): the control (saline treatment), untreated PCOS model, Heqi San treated PCOS model and metformin (MET) treated PCOS model.

### Heqi san or metformin treatment

The Heqi San was provided by the Traditional Chinese Medicine Hospital of Shenzhen (Shenzhen, China). The Heqi San formula is shown in Table 1. Its preparation was as follows: 25% weight of a mixture of *Schisandra chinensis (Turcz.) Baill.*, *Cynanchum otophyllum C. K. Schneid.,* and *Hordeum vulgare L.* was crushed into powder and sieved. After blending with other components, the mixture was dissolved in 30,000 ml ddH_2_O and heated for 1.5 h with a boiling heater, then the liquids were leached, 24,000 ml ddH_2_O was added into the dregs and reheated for 1.5 h, then the liquids were again leached, merged into an unguent with a relative density of 1.25–1.30, and were formed into pills by mixing with 1 g activated charcoal coat. In the control and untreated PCOS model groups, saline was used as control solution. In Heqi San treated PCOS (Heqi) and metformin (MET) treated PCOS groups, Heqi San or metformin was dissolved in saline and orally administered. The conversion of the equivalent drug dose from human to rats is based on body surface area (BSA), dose of drugs in rats = dose of drugs in human × (BSA of rats/ BSA of human) [25]. Heqi San was given at 8.1 g/kg by a cannula in PCOS rats, while metformin was given at 135 mg/kg in PCOS rats based on the previous study [26]. Animal were treated for 30 consecutive days.

**Table 1:** The formula of Heqi San

Number	Components	Weight (g)	Pharmacological effects
1	*Curculigo orchioides Gaertn.*	200	enhance natural immune function; inhibit thrombus formation; sedative effect; antibacterial activity
2	*Schisandra chinensis (Turcz.) Baill.*	200	improve nervous system cell function; promote the synthesis of cAMP, proteins, and glycogen; anti-stress effect; improve reproductive function
3	*Cynanchum otophyllum C. K. Schneid.*	200	regulate the innate immune system; enhance memory; protect the liver; act as an anti-inflammatory; endanger early pregnancy
4	*Citrus medica L. var. sarcodactylis Swingle*	200	calming effect in the central nervous system; expectorant action; anti-inflammatory effect; promote the secretion of digestive juices
5	*Crataegus pinnatifida Bunge*	600	reduce blood fat and blood pressure; antibacterial activity; scavenge free radicals; increase immune function
6	*Rhus chinensis Mill.*	180	antibacterial activity; pro-hemostasis; anti-tumor effect; improve myocardial ischemia
7	*Clinopodium megalanthum (Diels) C. Y. Wu & Hsuan ex H. W. Li*	300	used for the treatment of trichomonas vaginitis; sex hormone-like activities; anti-asthmatic; expectorant; antibacterial activity; anti-arrhythmic; anti-irritant; anti-osteoporotic; anti-inflammatory
8	*Cuscuta chinensis Lam.*	400	increase immune function; improve myocardial ischemia; improve sexual function; promote uterine health
9	*Poncirus trifoliata (L.) Raf.*	200	expectorant; digestive aid
10	*Hordeum vulgare L.*	1000	digestive aid; relieve breast pain; reduce blood sugar level; vasoconstrictor
11	*Polygala tenuifolia Willd.*	200	expectorant; sedative; promote uterine health; reduce blood pressure; antibacterial activity; anti-tumor effect
12	*Epimedium davidii Franch.*	200	Gonadotropin action; improve endocrine function; bidirectional regulatory effect on the immune system; reduce blood pressure; antibacterial activity; reduce blood sugar; treat erectile dysfunction

### Measurement of hormone levels

We used enzyme-linked immunosorbent assay (ELISA) kits (Cloud-Clone Corp., Houston, USA) to measure the serum concentrations of gonadotropins, including follicle stimulating hormone (FSH) and luteinizing hormone (LH), and steroid hormones, including 17β-estradiol (E2), progesterone (P), testosterone (T), and insulin. Insulin resistance was calculated to assess changes in insulin sensitivity according to the previous report [27].

### Histology

All rats were sacrificed after 30 days and the ovarian tissues were used for histology analysis. Ovarian tissues were fixed in 4% paraformaldehyde and embedded in paraffin. The sections were stained by hematoxylin and eosin (H&E) according to standard procedures. The ovary volume was measured and calculated using Image J software (NIH, USA).

### Real time quantitative PCR

Real-time quantitative PCR was used to measure the mRNA expression levels of GLUT4 and PTEN in the PI3K/AKT signaling pathway. Total RNA extraction was performed using TRIzol reagent (Life Technologies, USA) according to the manufacturer’s instruction. Two microgram of total RNA extracted from ovarian tissues was subjected to reverse transcription (RT) and cDNA synthesis was performed using a one-step RT-PCR kit from Takara (Takara, Japan). SYBR Green (Toyobo, Japan), RT-PCR amplification and real time fluorescence detection were performed using the ABI 7300 real-time PCR thermal cycle instrument (ABI, USA), according to the supplied protocol. The relative gene expression was calculated using the 2^-∆∆Ct^ method and the relative expression levels were normalized to the expression of endogenous GAPDH. The primers used in this study were: GLUT4 (F) 5′- GATCGGCTCTGAAGATGGGG-3′, GLUT4 (R) 5′- GGAGGAAATCATGCCACCCA-3′; PTEN (F) 5′-AGACCATAACCCACCACAGC-3′, PTEN (R) 5′-CAGGGCCTCTTGTGCCTTTA-3′; GAPDH (F) 5′-GGTATCGTGGAAGGACTCATGAC-3′, GAPDH (R) 5′-ATGCCAGTGAGCTTCCCGT TCAGC-3′.

### Western blotting

The protein expression levels of p-ERK, p-AKT, p-GSK3β, IRS-1, PTEN and GLTU4 was detected by western blotting. First, 2 μg tissue lysates were loaded on each lane of 10% polyacrylamide gel, and then blotted onto a polyvinylidene difluoride (PVDF) membrane. After blocking with a PBST containing 5% nonfat dry milk, the membrane was incubated with specific primary antibodies (Cell Signaling Technologies, USA). Peroxidase-linked IgG (Life Technologies) were used as secondary antibodies. These proteins were visualized with an ECL western blotting detection kit (Amersham Biosciences).

### Gene ontology analysis

The functions of the target genes were analyzed using bioinformatics. First, a gene ontology (GO) enrichment analysis was performed using DAVID (https://david.ncifcrf.gov/) [28]. The related pathways of the target genes were identified using the Kyoto Encyclopedia of Genes and Genomes (KEGG) databases.

### Statistical analysis

Data were presented as mean ± SEM. All the data were analyzed with Graphpad Prism 6.0. Paired Student’s t-tests and one-way ANOVA were used to determine significant differences. A *p* value less than 0.05 is considered to be significantly different.

## Results

### Heqi alleviated the disruption of serum hormone levels in PCOS model

**Fig. 1:**
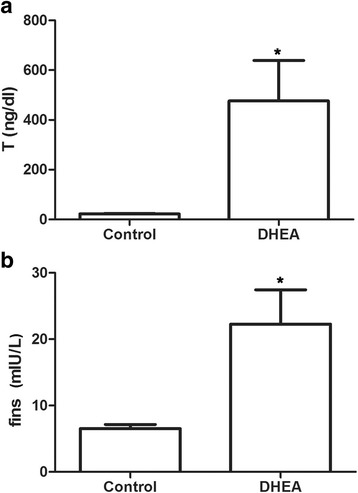
Establishment of PCOS model. **a** Testosterone concentration in serum was increased in PCOS model. **b** FINS level was elevated in PCOS model. *p < 0.05 vs. control

**Fig. 2:**
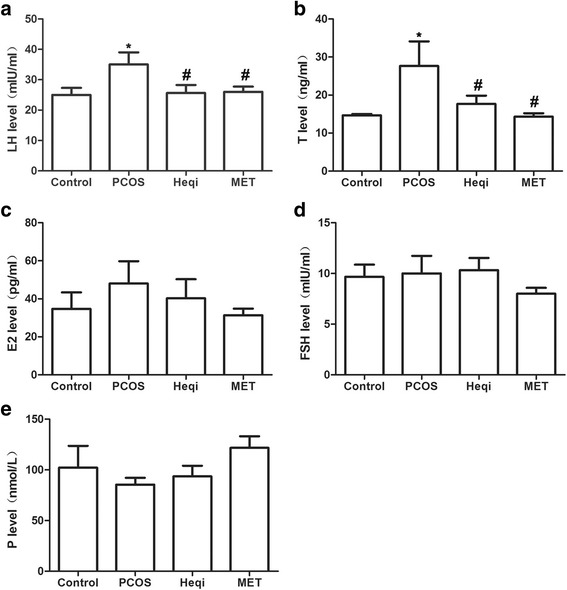
Application of Heqi San restored serum hormone levels in the PCOS model, including (**a**) luteinizing hormone (LH), (**b**) Testosterone (T), (**c**) estradiol (E2), and (**e**) follicule-stimulating hormone (FSH), but not (**e**) progesterone (P). *p < 0.05 vs. control, ^#^ *p* < 0.05 vs. PCOS

We first evaluated whether the PCOS model had been successfully established. We found that the serum testosterone (T) and fasting insulin (fins) levels increased after the DHEA injection when compared with control rats (Fig. 1a, b), demonstrating that the established model reflected the primary symptoms of polycystic ovary syndrome. Next, we determined whether the traditional Chinese medicine Heqi San had any effects on these symptoms. LH and T levels increased in the PCOS model group, while the application of metformin and Heqi were shown to counteract the increased LH and T levels (Fig. 2a, b). A similar result was obtained the level of E2. However, the alterations in E2 and P level were not statistically different (Fig. 2c, e). By contrast, we did not observe any change in FSH in either the PCOS model or the Heqi-treated animals (Fig. 2d). Collectively, these data indicate that the serum hormone level was disrupted in the PCOS model, while the application of Heqi San alleviated the alteration in hormone levels.

### Heqi changed the HOMA-IR and IRI in the PCOS model

**Fig. 3:**
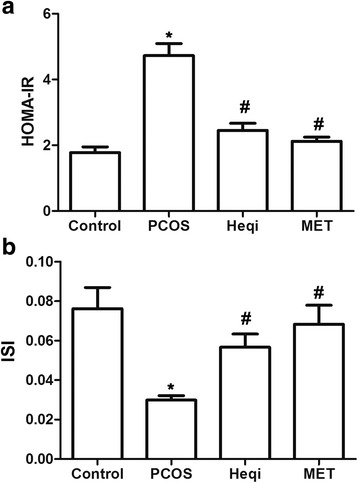
Heqi San treatment changed the insulin sensitivity in the PCOS model. **a** HOMA-IR was alleviated in both Heqi San-treated and MET-treated PCOS model. **b** Insulin sensitivity index (ISI) was decreased in the PCOS model, while Heqi San counteracted this effect. Heqi: Heqi San, MET: metformin. *p < 0.05 vs. control, ^#^ *p* < 0.05 vs. PCOS

We then determined whether or not the application of Heqi San could change the homeostasis model assessment-insulin resistance index (HOMA-IR) or the insulin sensitivity index (ISI) in the PCOS model. The HOMA-IR was increased in the PCOS model compared with that in control group (Fig. 3a). Both Heqi San and MET treatment were able to offset the increasing HOMA-IR in the PCOS model (Fig. 3a). In contrast, the ISI was significantly decreased in the PCOS model (Fig. 3b), Heqi San and MET were able to significantly increase the ISI level compared to that in the untreated PCOS model (Fig. 3b). These data demonstrate that Heqi San can antagonize the increasing HOMA-IR and the decreasing ISI in the PCOS model and that this effect is comparable to that of metformin.

### Heqi san leads to morphological recovery in the ovary

**Fig. 4:**
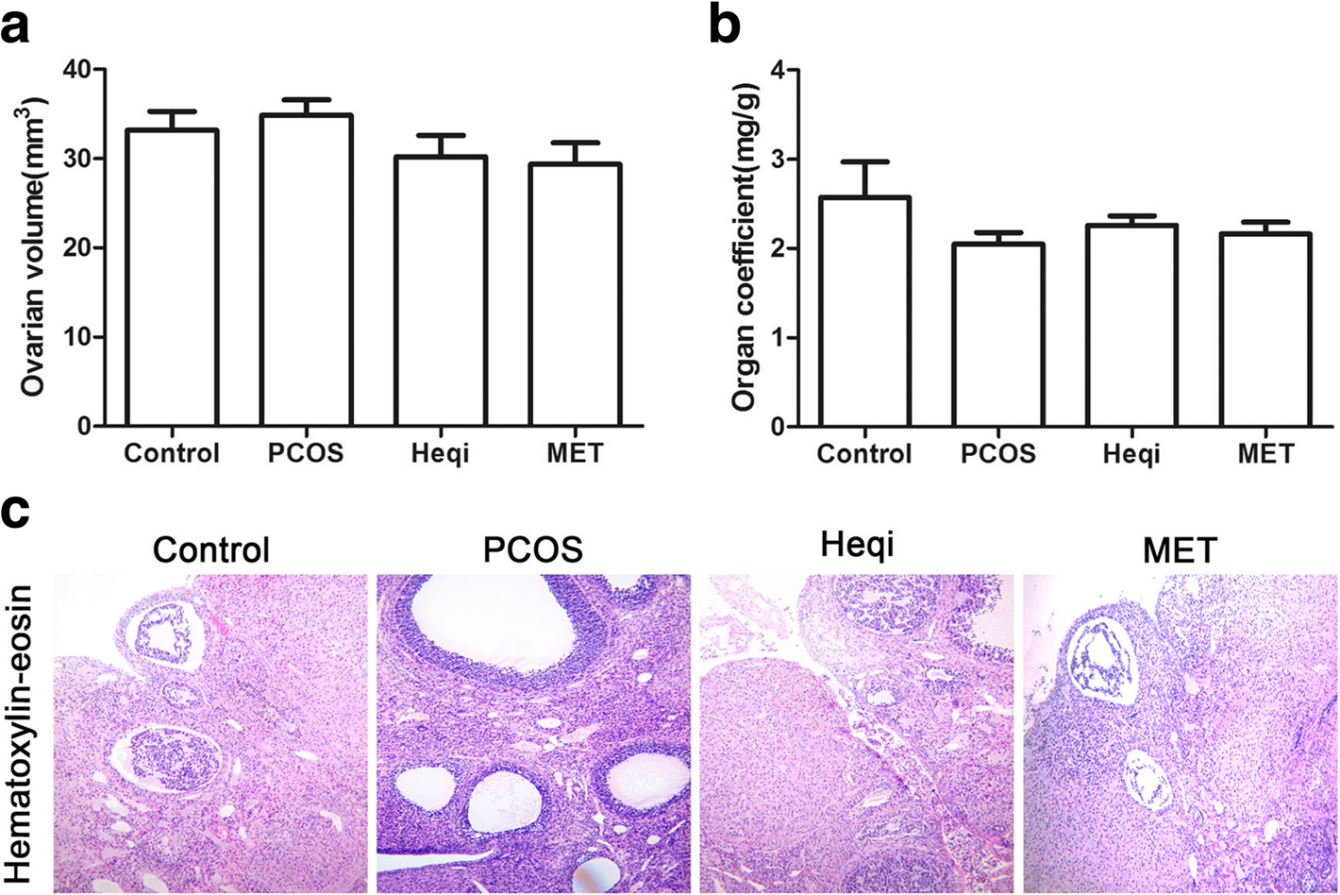
Morphological alteration in the ovary after Heqi San treatment. **a** Ovarian volume (mm^3^) was slightly changed after Heqi San treatment. **b** Organ coefficiency (mg/g) improved after Heqi San treatment in the PCOS model. **c** Compared with the control, the PCOS model showed apparent lesions in the ovary, Heqi San or MET treatment significantly restored the morphological damage in the PCOS model. Heqi: Heqi San, MET: metformin

Since Heqi San could modulate the serum hormone levels, we investigated whether it could restore the ovarian morphological changes in the PCOS model. We found that the ovarian volume was slightly increased in the PCOS model, but that the application of either Heqi San or MET decreased the volume (Fig. 4a). Meanwhile, the organ coefficient was reduced in the untreated PCOS model. However, the application of Heqi San or MET only slightly restored the organ coefficient (Fig. 4b). H&E staining showed ovarian follicles at different developmental stages in the control group (Fig. 4c). In contrast, the structure of ovarian tissue in the PCOS group was in a state of disorder, with apparent cystic dilatation in the ovarian follicles (Fig. 4c). Furthermore, the oocytes in the follicles disappeared and the number of granule cell layers decreased significantly (Fig. 4c). Upon treatment with either Heqi San or MET, the structure was partially recovered. As shown in the Fig. 4, the oocytes were once again found in follicles and the number of granule cell layers increased in both groups. Based on these data, we concluded that Heqi San was able to reverse the morphological disorder in ovarian tissues.

### Heqi restored the ovarian disorder through the PI3K/AKT pathway

**Fig. 5:**
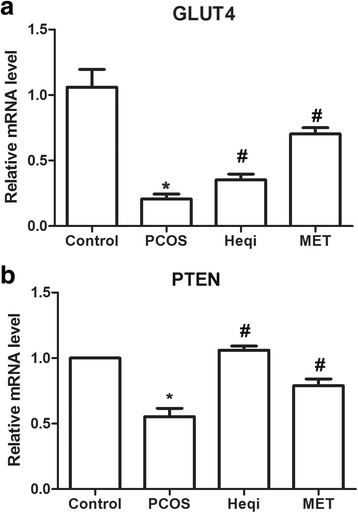
The effects of Heqi San on the PCOS model was mediated through the PI3K/AKT pathway. Heqi San treatment increased the expression levels of GLUT4 (**a**) or PTEN (**b**) in the PCOS model, where both of key signals were significantly down-regulated. Heqi: Heqi San, MET: metformin. *p < 0.05 vs. control, ^#^ *p* < 0.05 vs. PCOS

In order to confirm the involvement of the PI3K/AKT pathway in Heqi San-induced ovarian recovery in the PCOS model, we determined the expression alteration of key genes in the PI3K/AKT pathway, including GLUT4 and PTEN. The real-time quantitative PCR data demonstrated that GLUT4 and PTEN mRNA expression levels were significantly decreased in the PCOS model when compared with the control group (Fig. 5a, b), while the application of either Heqi San or MET drastically increased their expression levels (Fig. 5a, b). Those results indicate that the effects of Heqi San on PCOS are correlated with the PI3K/AKT pathway.

### Insulin resistance was alleviated with the treatment of Heqi san

**Fig. 6:**
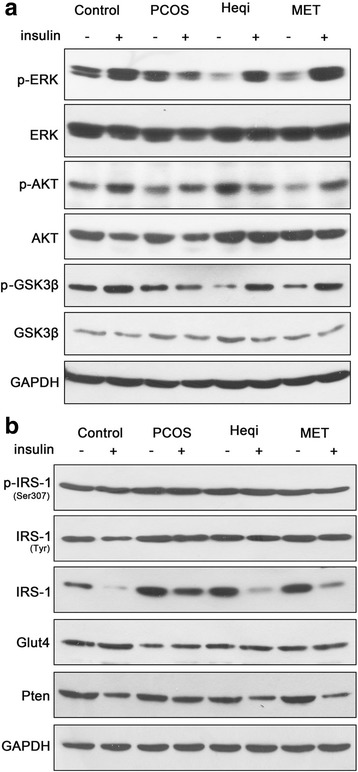
Insulin resistance was changed by Heqi San treatment in the PCOS model. **a** Treatment with either Heqi San or MET significantly improved the response to insulin stimulation, leading to the up-regulation of p-ERK, p-AKT, or GSK3β in the PCOS model. **b** Heqi San treatment recovered the decrease in IRS-1 and PTEN expression levels, which were inhibited in the PCOS model. Heqi: Heqi San, MET: metformin

Since insulin resistance is an important pathological feature in PCOS, we then checked whether Heqi San treatment could alleviate the insulin resistance in the PCOS model. In the control group, insulin stimulation increased the expression levels of phosphorylated ERK, AKT and GSK3β (Fig. 6a).Nevertheless, the significant increase was not found in the PCOS model after insulin stimulation. The insulin resistance was alleviated when either Heqi San or MET was applied. The expression levels of p-ERK and GSK3β were increased significantly after insulin stimulation (Fig. 6a). Furthermore, the expression levels of insulin receptor substrate-1 (IRS-1) and PTEN decreased significantly after insulin stimulation in the control group (Fig. 6b). In contrast, we did not observe any alteration in IRS −1 or PTEN expression levels in the insulin-treated PCOS model. Treatment with either Heqi San or MET decreased the expression levels of IRS-1 and PTEN in the insulin-treated PCOS model (Fig. 6b). However, we did not observe any alteration in GLUT4 or p-IRS-1 expression, indicating its irrelevance in insulin resistance. Collectively, these data demonstrate that Heqi San alleviated insulin resistance through the PI3K/AKT signaling pathway.

### Differential miRNA expression contributed to the pathological process in the PCOS model

**Fig. 7:**
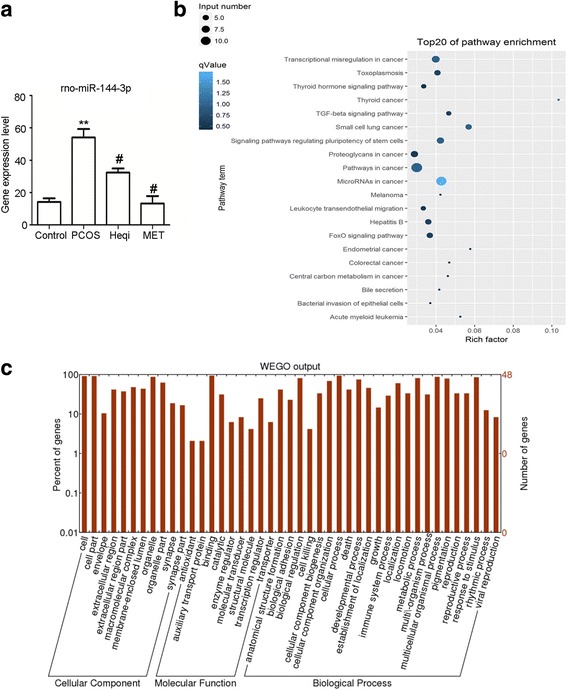
Analysis of the potential roles of miRNAs in PCOS pathogenesis. **a** The potential role of rno-miR-144-3p, the target gene of PTEN, was elucidated through real time PCR in the PCOS model and the Heqi-treated group. ^**^ *p* < 0.01 vs. control, ^#^ *p* < 0.05 vs. PCOS. **b** GO analysis disclosed the functions of target genes. **c** GO enrichment analysis demonstrated the signal pathways potentially involved in pathological mechanism of PCOS. Heqi: Heqi San, MET: metformin

We used sequencing and bioinformatics analysis to predict that PTEN was the target gene of rno-miR-144-3p (Table 2). Therefore, we identified the expression of rno-miR-144-3p in different groups, and found that its mRNA expression level was significantly increased in the PCOS model compared to the control (Fig. 7a). Treatment with either Heqi San or MET led to the decrease of the rno-miR-144-3p expression level in the PCOS model (Fig. 7a). This revealed that PTEN likely plays a critical role in PCOS pathology, and that it is regulated through rno-miR-144-3p. Furthermore, GO analysis indicated the target genes involved in PCOS pathology were mostly abundant in the category of binding and catalytic function. In terms of biological process, the functions of these target genes included response to stimulus, biological regulation, and cellular process, among others (Fig. 7b). The Go enrichment analysis also showed that the involved signaling pathways included the inflammation and apoptosis pathways (Fig. 7c).

**Table 2:** The prediction of target genes of miRNAs potential correlated with pathological mechanism of PCOS

miRNA ID	Target Gene
rno-miR-124-3p	Bdnf
rno-miR-124-3p	Itgb1
rno-miR-124-3p	Lamc1
rno-miR-124-3p	Neurod1
rno-miR-124-3p	Stat3
rno-miR-141-3p	Zeb2
rno-miR-142-5p	BTG3
rno-miR-144-3p	Celf2
rno-miR-144-3p	PTEN
rno-miR-151-5p	Fndc1

**Fig. 8:**
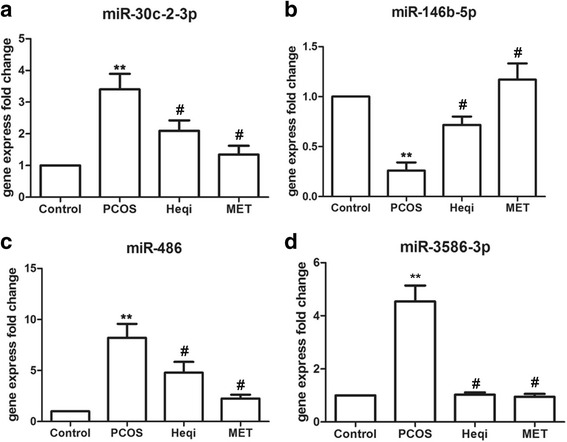
Differential expression levels of miRNA participating in PCOS pathogenesis, including miR-30c-2-3p (**a**), miR-146b-5p (**b**), miR-486 (**c**), and miR-3586-3p. **p < 0.01 vs. control, ^#^ *p* < 0.05 vs. PCOS

We then used the bioinformatics analysis to identify certain differences in miRNA between the control group and PCOS groups, and some of the identified miRNAs involved in the P13K/AKT pathway are listed in Table 3. Accordingly, we selected four miRNAs (rno-miR-30c-2-3p, rno-miR-146b-5p, rno-miR-486 and rno-miR-3586-3p) and determined their expression levels in the different groups. The primers used for the miRNAs expression analysis are shown in Table 4. The real time quantitative PCR results indicated that the expression levels of rno-miR-30c-2-3p, rno-miR-486 or rno-miR-3586-3p were significantly higher in the PCOS model (Fig. 8a–d), while Heqi San or MET treatment could decreased the expression levels of all three miRNAs when compared with the untreated PCOS group (Fig. 8a–d). In contrast, the expression level of rno-miR-146b-5p was decreased in the PCOS model (Fig. 8b), and treatment with either Heqi San or MET increased its expression level (Fig. 8b). These results are consistent with our sequencing data, indicating the critical role of the PI3K/AKT pathway in the pathology of PCOS in our model.

**Table 3:** Lists of miRNAs involved in PI3K/AKT pathway

mRNA	protein	gene ID	control-Expression	PCOS-Expression	Up-Down-Regulation (contol/NPCOS)	*p*-value
PTEN	PTEN	miR-494-3p	75	36	Up	0.1878728
PTEN	PTEN	miR-132-3p	353	163	Up	0.0199686
Slc2a4	GLUT4	miR-133a-3p	140	49	Down	0.7730520
Slc2a4	GLUT4	miR-133b-3p	97	46	Up	0.1579726
PTEN	PTEN	miR-144-3p	260	423	Up	0.0000000
PTEN	PTEN	miR-212-3p	66	27	Down	0.6160000
Rela	NFkB	miR-21-5p	170,370	45,208	Down	0.0000000
PTEN	PTEN	miR-216a-5p	17	7	Down	0.7146180
PTEN	PTEN	miR-26a-5p	206,732	46,330	Down	0.0000000
PTEN	PTEN	miR-26b-5p	9174	3703	Down	0.0000100
Mapk1	Mapk	miR-290	3	3	Up	0.1814094
Rela	NFkB	miR-29a-3p	14,732	9573	Up	0.0000000

**Table 4:** The primers used for miRNAs expression analysis

Name	primers sequences (5′-3′)
rno-miR-30c-2-3p	F: GCTGGGAGAAGGCTGTT
	R:TGTCGTATCCAGTGCAGGGTCCGAGGTATTCGCACTGGATACGACAGAGTA
rno-miR-146b-5p	F: GGGTGAGAACTGAATTCCA
	R:TGTCGTATCCAGTGCAGGGTCCGAGGTATTCGCACTGGATACGACACAGCC
rno-miR-486	F: GGGGATACTAGACTGTGAGCT
	R:TGTCGTATCCAGTGCAGGGTCCGAGGTATTCGCACTGGATACGACTCGAGG
GAPDH	F: GGTATCGTGGAAGGACTCATGAC
	R: ATGCCAGTGAGCTTCCCGT TCAGC

## Discussion

In this study, we elucidated the beneficial effects of Heqi San for PCOS through the establishment of a disease model in rats. We found that treatment with Heqi San alleviated the serum hormone imbalance and improved insulin resistance. This effect was comparable with that of the widely used medicine metformin and thus shows great promise in the application of Heqi San for PCOS treatment. Furthermore, its potential effects are likely mediated by the PI3K/AKT pathway. PCOS has several clinical manifestations, mainly including chaotic hormone levels such as with LH and androgens, and increasing insulin resistance [29]. Therefore, any medicine that can safely improve the above symptoms would be an ideal candidate for PCOS therapy. However, not all PCOS patients are suitable for metformin treatment [30]. TCM can be used as a dietary herbal supplement for PCOS treatment in order to decrease the side effects of some western medicine.

In this study, we found that Heqi San has both functions. Firstly, it can regulate the serum hormone levels in PCOS. It can regulate gonadotropins including follicle stimulating hormone and luteinizing hormone, as well as steroid hormones including 17β-estradiol, progesterone, and testosterone. TCM consists of a large number of herbal medicines, which may regulate hormone levels. Some components have a two-way regulating effect on hormone levels through compatibility effects [31]. A major characteristic of PCOS is an elevated serum androgen level; orally administered Heqi San could reduce the abnormal secretion of androgen and achieve a physiological androgen balance. It contains a variety of components, such as *Epimedium davidii Franch.* and *Cuscuta chinensis Lam.*, which can regulate the sex gland-adrenal gland axis to reduce the androgen level and induce ovulation [32]. We speculate these action are probably due to its effect on the androgen receptor (AR), thereby regulating androgens-gonadotropin interactions [33]. Secondly, Heqi San can improve insulin resistance, which is most likely due to its regulation of the correlation between insulin resistance and thyroid-stimulating hormone. Insulin resistance and the dysregulation of glucose metabolism are common in PCOS patients [34]. The effect of Heqi San on insulin resistance may also be due to an improvement in beta cell function, which is directly responsible for insulin secretion [35]. As two of the components of Heqi San, *Schisandra chinensis* can promote the synthesis of cAMP, proteins, and glycogen and can enhance glycogen metabolism [36] and *Polygala tenuifolia Willd.* protected diabetic rats from hippocampal injury [37]. In addition, many PCOS patients are obese, which may cause sugar metabolism disorders and pregnancy complications [38]. *Poncirus trifoliata (L.) Raf.*, *Hordeum vulgare L.,* and *Crataegus pinnatifida Bunge* have been shown to aid digestion, reduce blood sugar levels, and may even help with weight loss [39–41].

One of the important findings in this work was the involvement of the PI3K/AKT pathway in the Heqi San-induced beneficial effects on PCOS. The role of the PI3K/AKT signaling pathway in PCOS pathogenesis has been reported previously, and is probably due to the fact that the activation of AKT leads to the enhanced insulin resistance [42]. Several important elements in the PI3K/AKT pathway were regulated in our rat PCOS model by insulin stimulation, a process potentially modifiable by Heqi San treatment. These results are consistent with the previous finding that PI3K inhibition has beneficial effects on PCOS [43]. This beneficial effect lead to improvements in insulin, testosterone, and luteinising hormone levels, and is comparable with the findings of the current study. Since phosphorylation of ERK, AKT and GSK3βis significantly affected in insulin resistance after Heqi San treatment, further work will be focused on the pharmaceutical inhibition of phosphorylation in key signals of the PI3K/AKT pathway.

Through the bioinformatics analysis, we found that there were certain miRNAs that seemed to play important roles in Heqi San-induced therapeutic effect on the PCOS model. Previous work has demonstrated that expression of miRNAs in the uterus of a rat PCOS model was significantly altered [44]. This is consistent with the general concept that the abnormal expression of miRNAs is correlated with various disorders [45]. Through the same bioinformatics work, we predicted several miRNAs that were differentially expressed between the control and treatment groups, and found that their target genes are important factors in the PI3K/AKT pathway. In fact, miRNAs have been reported to regulate cellular processes, such as cell proliferation and differentiation [46]. Combined with the GO analysis, our prediction indicated that some miRNAs may regulate proliferation, differentiation, and apoptosis, correlating with PCOS pathogenesis. To prove this hypothesis, direct genetic manipulation of these miRNAs will be conducted in PCOS models, in order to provide evidence to clarify their role in the pathogenesis of PCOS.

## Conclusion

In summary, we reported on the beneficial effects of Heqi San in a PCOS rat model, including improvements in serum hormone levels, ovarian morphological recovery and insulin resistance. This effect is likely mediated by the PI3K/AKT pathway. We also identified important miRNAs that are potential involved in the therapeutic effect of Heqi San and in the pathogenesis of PCOS. This work provides experimental evidence to support the potential application of Heqi San, putting forward a new methodology for PCOS treatment.

## Abbreviations

AR: Androgen receptor
E2: 17β-estradiol
ELISA: Enzyme-linked immunosorbent assay
Fins: Fasting insulin
FSH: Follicle stimulating hormone
GO: Gene ontology
H&E: Hematoxylin and eosin
HOMA-IR: Homeostasis model assessment-insulin resistance index
ISI: Insulin sensitivity index
KEGG: Kyoto encyclopedia of genes and genomes
LH: Luteinizing hormone
MET: Metformin
P: Progesterone
PCOS: Polycystic ovary syndrome
PVDF: Polyvinylidene difluoride
T: Testosterone
